# Frequency-noise measurements of optical frequency combs by multiple fringe-side discriminator

**DOI:** 10.1038/srep16338

**Published:** 2015-11-09

**Authors:** Nicola Coluccelli, Marco Cassinerio, Alessio Gambetta, Paolo Laporta, Gianluca Galzerano

**Affiliations:** 1 Dipartimento di Fisica - Politecnico di Milano, Piazza Leonardo da Vinci 32, 20133 Milano, Italy; 2 Istituto di Fotonica e Nanotecnologie - CNR, Piazza Leonardo da Vinci 32, 20133 Milano, Italy

## Abstract

The frequency noise of an optical frequency comb is routinely measured through the hetherodyne beat of one comb tooth against a stable continuous-wave laser. After frequency-to-voltage conversion, the beatnote is sent to a spectrum analyzer to retrive the power spectral density of the frequency noise. Because narrow-linewidth continuous-wave lasers are available only at certain wavelengths, heterodyning the comb tooth can be challenging. We present a new technique for direct characterization of the frequency noise of an optical frequency comb, requiring no supplementary reference lasers and easily applicable in all spectral regions from the terahertz to the ultraviolet. The technique is based on the combination of a low finesse Fabry-Perot resonator and the so-called “fringe-side locking” method, usually adopted to characterize the spectral purity of single-frequency lasers, here generalized to optical frequency combs. The effectiveness of this technique is demonstrated with an Er-fiber comb source across the wavelength range from 1 to 2 *μ*m.

In the last fifteen years, optical frequency combs (OFCs) have attracted huge interest among the scientific and industrial research community, owing to their unique combination of broad bandwidth, low phase and amplitude noises, and absolute frequency calibration in a single device. Originally developed for metrological applications, i.e. highly accurate measurements of optical frequencies^1^,^2^,^3^, OFCs have had deep impact in many other research areas such as attosecond science^4^, high-resolution spectroscopy^5^,^6^,^7^,^8^,^9^, optical waveform generation^10^, remote sensing and distance measurements^11^,^12^, low-phase-noise microwave synthesis^13^,^14^, optical communications^15^, and astrophysics^16^. Presently, commercial OFC systems cover the visible and near-IR spectral region, whereas laboratory systems reach the terahertz^17^,^18^, as well as the mid-IR^19^,^20^,^21^,^22^,^23^ and extreme-ultraviolet^24^,^25^ regions.

The OFC can be seen as an array of thousands phase-coherent single-frequency lasers emitting at discrete and evenly spaced frequencies (comb teeth). The optical frequency *v*_*p*_ of each comb tooth is defined by three parameters, namely the tooth order *p* (typically of order 10^5^–10^6^), the comb frequency spacing *f*_*r*_, and the offset frequency *f*_*o*_, through the simple relation *v*_*p*_ = *pf*_*r*_ + *f*_*o*_. Typically, the comb teeth spacing 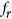 (comb repetition rate) ranges from tens of MHz up to tens of GHz according to the femtosecond laser resonator configuration^26^, and reaches even hundreds of GHz in microring combs^27^,^28^. The comb offset frequency 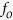 is within the range from 0 Hz to 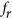.

The ultimate limit of all the precision measurements making use of OFCs is represented by the frequency noise (FN) of the comb teeth and hence their linewidth. For this reason, the FN of OFCs has been widely investigated to identify the main noise contributions^29^, and different active stabilization techniques have been implemented to narrow the comb teeth linewidth. To this purpose, both the 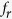 and 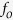 frequencies have to be stabilized against a frequency reference: microwave frequency standards (H-maser or Cs-clock) and slow feedback loop can effectively improve the OFC long term stability^1^, whereas ultra-narrow linewidth optical frequency standards (Hz-level laser stabilized against Ultra-Low-Expansion cavity, or optical clock) and fast servo electronics can further narrow the comb teeth relative linewidth down to the mHz-level across the full spectral range of the OFC^30^,^31^. Considering the growing impact of OFCs on current research and their application as frequency rulers, it is apparent that the characterization of OFCs in terms of FN is a fundamental prerequisite.

A time-domain approach is typically adopted to measure the FN of an OFC, based on hetherodyning one comb tooth against a high-spectral purity reference laser. As a necessary condition, the FN of the reference laser has to be well below the FN of the comb tooth under test. The frequency fluctuations of the hetherodyne beat are then converted into voltage fluctuations by a phase-locked voltage-controlled oscillator, providing a signal proportional to the FN of the comb tooth^32^. The limitations of this approach are apparent, as the measurement of the FN across the broad spectral region typically covered by the comb requires a set of reference lasers operating around the different wavelengths of interest^33^; this limitation becomes even stronger when considering the recent progress in comb technology, that extended the spectral coverage into the UV, mid-IR and THz regions, where narrow-linewidth reference lasers are not easily available. This approach has been also extended to simultaneously measure the FN of many teeth over the emission spectrum of the comb under test, using a second and identical comb operating at a slightly detuned repetition rate for parallel hetherodyning of the comb teeth^34^. The main drawback of this technique is the need for two OFC systems.

By contrast, frequency domain approaches, based on optical frequency discriminators, are more general and do not require supplementary reference lasers. The FN is directly converted and acquired as a voltage fluctuation by a low-noise photodiode, without the need for a phase-locked oscillator. Fabry-Perot (FP) resonators, with a proper linewidth as compared to the oscillator noise bandwidth, can be employed to perform high-sensitivity measurements of the FN of CW lasers regardless of the emission spectral region^35^,^36^.

In this paper, we report on a direct method for the measurement of the FN of OFCs based on a low-finesse FP resonator operating as an array of optical frequency discriminators. The method is a generalization of the fringe-side locking technique^35^, as commonly used for the characterization of single-mode CW lasers, and allows for FN measurement across the entire emission spectrum of the OFC without using supplementary reference lasers. Although the combined use of OFCs and passive cavities has been widely investigated^7^,^9^,^25^, to the best of the Authors’ knowledge, this approach has never been used for the FN of OFCs. In principle, the proposed technique allows for FN measurements in any spectral region from the UV to the mid-IR by simply selecting proper FP mirrors; extension to the THz would be also feasible provided that mirrors with specified reflectivities would be available in this wavelength region. As the spectral coverage of OFCs can be very wide, reaching even an octave, band-pass filtering of the OFC can be necessary for the application of the proposed technique to reduce the band under investigation to a span compatible with the FP mirror reflectivity. The measurement of the FN within relatively narrow sub-bands allows for exploration of the FN behavior across the much broader comb bandwidth. The proof of concept given in this paper is based on the characterization of the FN of a commercial Er-fiber frequency comb in the spectral range from 1 to 2 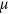
m, demonstrating the great potential and flexibility of the proposed method.

## Theory

**Figure 1:**
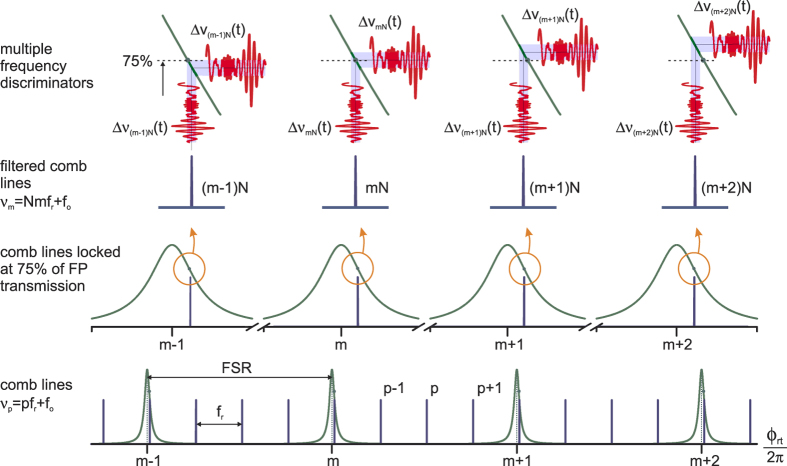
Principle of the multiple frequency discriminators for FN measurement of OFC. The comb lines at frequency 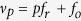 are pre-filtered with a filtering factor 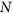 using a low-finesse Fabry-Perot cavity with free spectral range 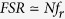. Cavity resonances are labeled by order 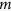, corresponding to the round-trip phase shift of pulses expressed as integer multiple of 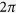. A slow feedback loop keeps the overall Fabry-Perot transmission locked to 75% of the maximum. This implies that each filtered comb line of order 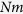 is weakly locked to the side of the corresponding resonance of order 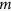, and is probed with similar slopes. The exact operating point of each single frequency discriminator (i.e. Fabry-Perot resonance) around the nominal 75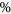-level is slightly different mainly because efficient comb-cavity coupling in the presence of non-zero offset frequency 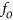 requires 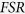 detuned from 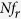.

The operating principle of the proposed technique is shown in Fig. 1. The comb under investigation, whose output spectrum is constituted by the frequency components 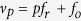, is coupled to a FP cavity with proper width of the resonances (10–100× the expected linewidth of the comb tooth). In general, the free spectral range (FSR) of a FP cavity is frequency-dependent because of the dispersion originating from cavity mirror coatings^37^,^38^. The effect of dispersion is particularly relevant when working with high-finesse cavities or near the edges of the mirror reflectivity. For the sake of simplicity, we will neglect the effect of dispersion in the calculation that follow. However, this can be considered a good approximation when working with a FP cavity with low finesse of ~100, and mirror reflectivity broader than the comb bandwidth. Under these assumptions, the FP resonant frequencies are simply given by 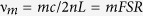, where 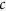 is the speed of light in vacuum, 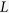 is the FP length, n ≃ 1 is refractive index of air, and 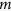 is the fringe order.

A maximum of the FP transmission is obtained in the “optimal resonance condition”, represented by the equation


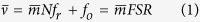


where 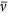 is the frequency of the comb tooth nearest to the center of the comb spectrum and 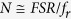 the filtering ratio (Fig. 1 shows an example for 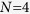). In the resonance condition, the FP cavity selects a subset of comb teeth (one every 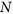), however, because the offset frequency 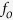 is typically non-zero, this implies 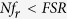, and hence each subsequent FP resonance away from the center frequency 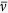 has an additional detuning 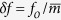 with respect to the corresponding comb mode. All the comb modes out of the FP resonances, characterized by orders 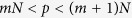, are sufficiently rejected even using cavity finesse of 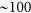 (attenuation >20 dB for N = 4), so that their contribution to the FN measurement is negligible. The FP acts as a frequency discriminator when its transmission is tuned at around 75% of the maximum, that is in the operating region of highest linearity of the FP transmission fringe (Airy function). Under these circumstances, the comb tooth at 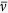 operates at the nominal 75% transmission point, whereas the comb teeth at lower (higher) frequencies operate at transmission decreasing (increasing) proportionally to the frequency detuning with respect to the corresponding FP resonance. On the other hand, the fringe slopes probed by each comb tooth are very similar, provided that all the teeth up to the edges of the comb spectrum operate within a certain limited region around the 75% transmission point. To this purpose, the number of comb teeth transmitted by the FP cavity is assumed to be 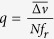, where 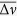 is the FWHM of the filtered comb spectrum. The cumulative frequency detuning of the FP resonances over the comb band is


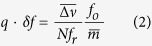


The FP fringe has high slope (sensitivity) and good linearity in the region from approximately 55% to 85% of the maximum transmission, corresponding to nearly a quarter of the FP resonance bandwidth 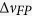 (FWHM). Operation within this desirable region requires


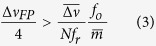


Once this condition is met, the frequency fluctuations of each comb tooth filtered by the FP cavity are converted into amplitude fluctuations that can be measured by a low noise photodetector (see Fig. 1), representing a generalization of the standard fringe side-locking technique adopted for the characterization of the FN of single-frequency CW lasers^35^.

The comb intensity noise contribution can be efficiently rejected using a balanced detection scheme^39^, yielding a highly-sensitive FN measurement where all the filtered comb teeth coherently combine with similar frequency-to-voltage conversion coefficients. Assuming that each filtered comb tooth gives a voltage contribution on the photodiode at the output of the FP cavity 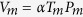, where 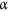 is the power-to-voltage conversion coefficient of the photodiode, 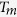 is the FP transmission at the optical frequency 
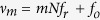
 and 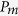 is the power of the 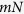-th comb tooth, the voltage fluctuations due to intensity and frequency noise can be expressed as 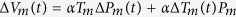, where 
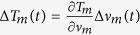
, the product 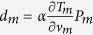 represents the frequency-to-voltage coefficient of the single fringe at the mN-th comb tooth, and 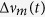 is the corresponding frequency fluctuation. The total voltage generated by the photodiode at the output of the FP cavity, 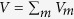, is given by the sum of the voltage contributions of all the comb teeth transmitted by the FP cavity. The corresponding voltage fluctuations are given by


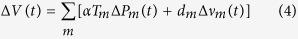


where the first and second term in the sum represent the comb intensity and frequency noise, respectively. By neglecting the intensity noise that is rejected using a balanced detection scheme and assuming that each comb tooth within the band of interest is characterized by the same FN, the resulting balanced voltage fluctuations are


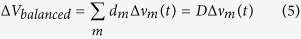


where 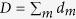 represents the overall FP frequency-to-voltage discrimination coefficient. In the presence of dispersion effects, the coefficients 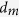 in the summation are slightly changed and accounted for into the resulting value of 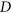, hence the calculations presented are still valid. The FN power spectral density (PSD) of the comb, 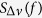 in unit of Hz^2^/Hz, can be easily obtained from the PSD of the signal at the output of the balanced detector using the relation 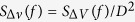.

The selection of appropriate FP mirrors is governed by few simple guidelines. In general, FP mirrors with lower finesse are preferable to fulfill Eq. (3), that is necessary to get correct FN measurement. However, lower finesse implies lower sensitivity. Hence, the general rule is to select the lowest finesse compatible with the required noise floor of the setup. The noise floor 
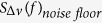
, i.e. the minimum FN PSD detectable, is dominated by the noise at the output of the balanced detector. The photodetector noise is usually expressed in terms of Noise-Equivalent-Power (NEP) in units of W/Hz^1/2^, that is the power of light impinging on the detector which produces a photocurrent equal to the noise level. The minimum FN PSD 
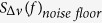
 can be obtained from the NEP using the formula 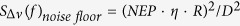 where 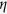 is the responsivity of the detector in units of A/W, 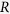 is the impedence of the electrical spectrum analyzer employed for the measurement of the FN PSD, and 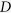 is the frequency-to-voltage discrimination coefficient. Trivially, the noise floor has to be much lower than the FN PSD of the OFC under investigation. In many applications, the requirements are only expressed in terms of linewidth, a simplified parameter representing an integrated measurement of the FN PSD. In these cases, the integral of the noise floor over the bandwidth of interest, limited to the Fourier frequency range where the noise floor lies above the beta-line^41^, that is the only range contributing to the linewidth, represents the minimum measurable linewidth. Similarly to the FN PSD, the minimum measurable linewidth has to be much lower than the linewidth of the OFC under investigation.

## Results and Discussion

### Frequency noise measurement of an Er:fiber OFC

**Figure 2:**
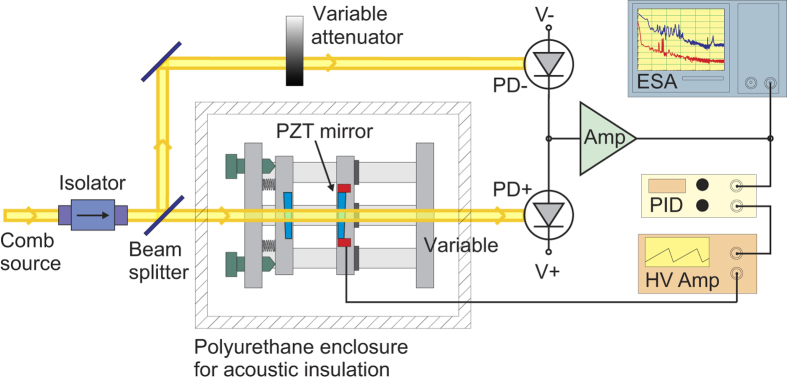
Schematic of the setup for linewidth measurement of comb tooth. PZT: piezo-electric transducer; PD: photodiode; Amp: high-bandwidth electrical amplifier; HV Amp: high-voltage amplifier; PID: proportional-integral-derivative controller; ESA: electrical spectrum analyzer.

**Figure 3:**
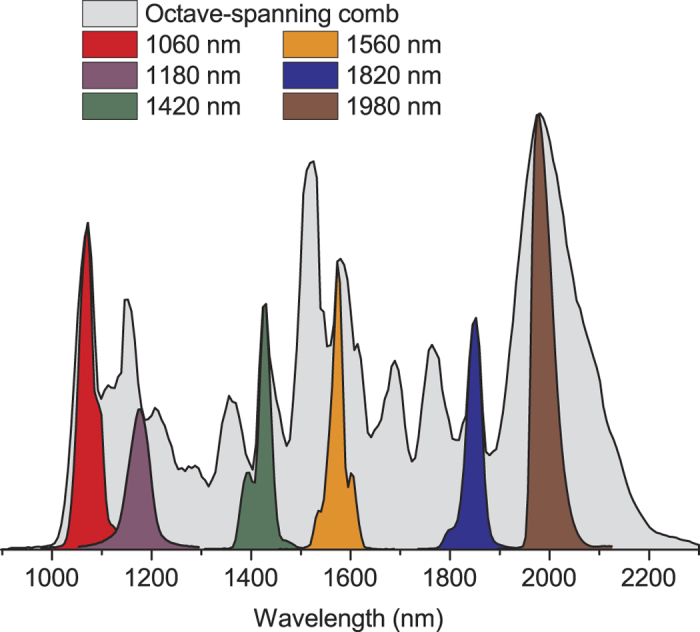


**Figure 4:**
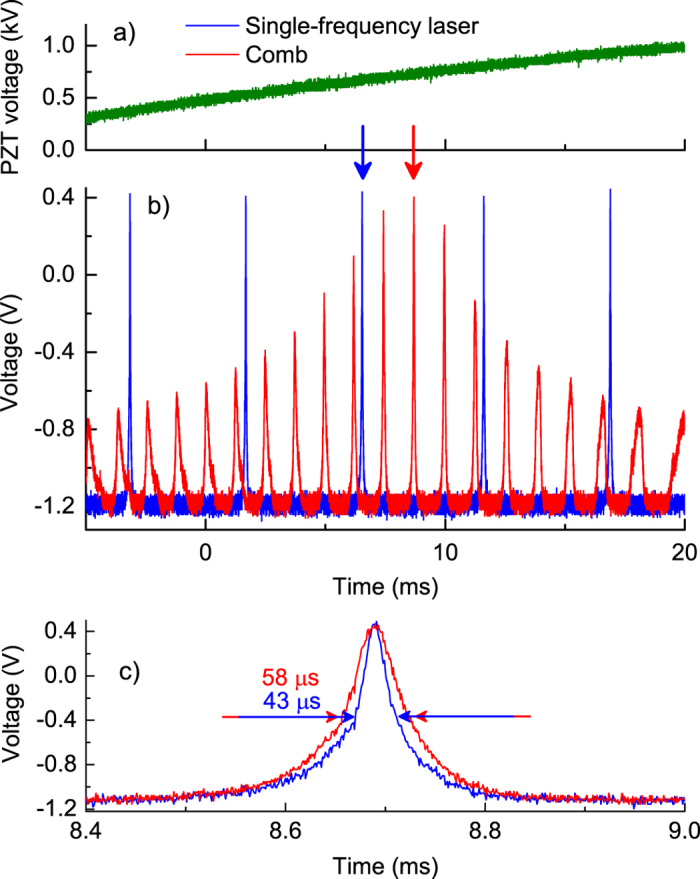
(**a**) Voltage applied to the PZT of the scanning FP. (**b**) Transmission of the scanning FP injected by the comb (red line) and single-frequency laser (blue line) as measured by balanced detection scheme. (**c**) Expanded view of resonances of the comb (main peak) and the single-frequency laser (translated for comparison) as indicated by the arrows.

The multiple fringe-side locking technique has been used to characterize the FN of an octave spanning Er:fiber OFC with power of 250 mW, repetition frequency 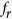 = 250 MHz, and spectral coverage from 1 to 2 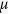
m. Figure 2 shows a schematic of the experimental setup. The OFC spectrum is narrowed by bandpass filters with central wavelength of 1060, 1180, 1420, 1560, 1820, or 1980 nm, and optical bandwidth of ~40 nm. The six resulting spectra, each with power content around 10 mW, are shown in Fig. 3. After spectral filtering, the comb passes through an optical isolator and a FP interferometer placed in a polyurethane enclosure to reduce acoustic perturbations and the effect of air turbulence. The frame of the FP (Burleigh RC-110) is made of Super-Invar (thermal expansion coefficient 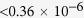) to guarantee an improved stability; previous tests to characterize high spectral-purity lasers have shown an upper limit on the linewidth contribution, due to the FP cavity, of 4 and 17 kHz over observation times of 1 and 100 ms, respectively^40^. When using the filter at 1560 nm, the FP cavity has a free-spectral-range (FSR) of 1 GHz and mirrors with nominal reflectivity of 97%, corresponding to a finesse of ~100 and a linewidth of ~10 MHz; similar FP characteristics have been used with the other bandpass filters. A balanced detection scheme is used to cancel the laser intensity noise contribution to the FN. The FP cavity transmission is kept at 75% of its maximum value by a servo-loop acting on the PZT actuator of the FP interferometer with a slow control bandwidth of ~1 Hz. The PSD of the signal at the output of the balanced photodetector corresponds to the comb frequency fluctuations for Fourier frequencies larger than the control bandwidth. Figure 4 shows the transmission of the scanning FP that is illuminated by either a single-frequency laser at 1560 nm (blue line) or a comb at 1560 ± 20 nm (red line). In the case of the single-frequency laser, the FP transmission is characterized by a classical Airy function with a periodicity of 4.4 ms, corresponding to the FSR of 1 GHz. A more complex structure is observed with the comb: as a first point, because the comb tooth frequency spacing 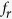 is 250 MHz, the FP transmission has 4 resonances within each FSR; as a second point, the envelope of the resonances has a maximum when the optimal resonance condition is reached (highest resonance), and an amplitude decreasing rapidly away from the optimal resonance, as the number of comb teeth coupled into the FP cavity is proportionally decreased; finally, the width of the resonances increases away from the optimal resonance, due to the increasing mismatch between FP resonances and comb teeth periodicity^9^. A detailed view of the highest FP resonance is reported in Fig. 4c). In the case of single-frequency laser, the cavity resonance has a linewidth of 43 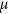
s (corresponding to 10 MHz) and a slope of 0.18 V/MHz at 75% of the maximum transmission. On the other hand, the 40-nm wide OFC radiation shows a slightly increased linewidth of 58 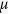
s and a reduced slope of 0.13 V/MHz, representing the overall frequency-to-voltage coefficient, ascribed to the non-zero comb offset frequency and hence to the detuning between cavity resonances and comb teeth. Under this condition, the slope 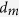 of the resonances probed by each comb tooth away from the 75% operating point is slightly reduced, as is the resulting overall slope 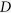 of the highest resonance. The minor increase of the FP linewidth when analyzing the OFC is already an indication that almost all teeth within the observed band are coupled into the FP cavity close to the 75% operating point. More specifically, the detuning is acceptable if the condition (3) is satisfied. The order of the tooth nearest to the center of the comb spectrum is 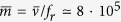. Assuming a comb bandwidth 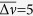 THz (40 nm), the number of comb teeth transmitted by the FP cavity is 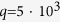. The highest comb offset frequency is 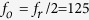 MHz, yielding the largest frequency detuning 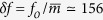 Hz. The resulting overall detuning over the band of the filtered comb is 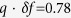 MHz, that is less than 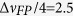 MHz, hence the condition (3) is easily satisfied. Similar conditions have been obtained in all other spectral regions analyzed.

**Figure 5:**
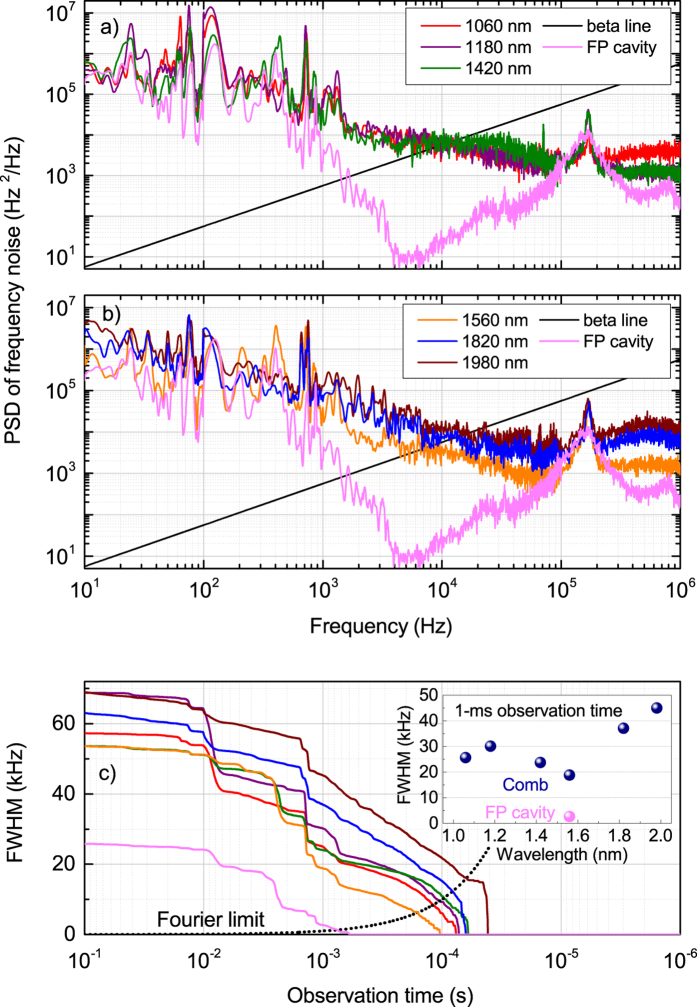
(**a,b**) PSD of FN of the filtered comb spectra and the FP cavity. (**c**) Linewidth of the filtered comb spectra and the FP cavity as a function of observation time; the inset shows the linewidth across the comb spectrum at 1-ms observation time.

The resulting FN PSDs of the bandpass filtered OFC at different wavelengths (1060, 1180, 1420, 1560, 1820, 1980 nm) are shown in Fig. 5 together with the 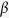-line, 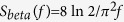
41 and the FN PSD of the FP cavity. In agreement with theoretical analysis of low-noise fiber-laser frequency combs reported in^29^,^42^, the comb teeth around 1560 nm show the minimum FN PSD. Due to environmental noise acting on the cavity length of the comb oscillator, this PSD falls off roughly as 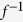 reaching a minimum value of 10^3^ Hz^2^/Hz at 50 kHz (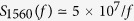 Hz^2^/Hz). For Fourier frequencies larger than 100 kHz the PSD is limited by the peak of the relaxation oscillations at ~200 kHz. Similar trends are observed in the other spectral regions of the comb, although additional noise contributions arise. In particular, the FN of the comb teeth around 1420, 1180, and 1060 nm, shows a pump-induced noise contribution^44^ over the 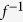 trend that limits the PSD in the Fourier frequency range from 2 to 100 kHz with a characteristic low-pass frequency cutoff at ~20 kHz. On the other hand, the FN around 1820 and 1980 nm can be fitted by the 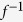 trend as 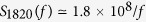 and 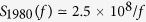 Hz^2^/Hz at Fourier frequencies below 100 kHz, whereas the excess noise generated during supercontinuum formation^43^ gives an 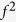 trend at Fourier frequencies above 100 kHz.

The FN PSD of the FP cavity has been measured using a high-spectral purity Er:fiber laser stabilized against a monolithic Ultra-Low-Expansion cavity. The resulting linewidth of the stabilized laser is below 100 Hz over 1-s observation time, i.e. much less than the linewidth of the FP cavity. Under this condition, the measurement of the FN of the stabilized laser by locking to the side of a FP fringe represents essentially a measurement of the FN PSD of the FP cavity at Fourier frequencies below 10 kHz; at higher Fourier frequencies, the servo bump centered at 180 kHz of the cavity-stabilized laser dominate the measurement of the FN PSD, however the resulting FP linewidth is not affected by this contribution because it lies below the 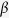-line^41^. As shown in Fig. 5(a,b), the FN PSD contribution of the FP cavity is lower than that of the comb in all the frequency range of acoustic vibrations below 10 kHz; however, the resonances below 1 kHz in the FN PSDs of the filtered comb are actually ascribed to the FP itself, and originates from vibration-induced cavity length changes.

Figure 5(c) shows the linewidth of the comb and FP cavity, calculated from the measured FN PSD by the *β*-line method proposed in^41^, as a function of the observation times. At 1-ms observation time, a minimum linewidth of 19 kHz is found for the comb teeth around 1560 nm. The linewidths across the comb spectrum are mainly broadened by the pump-induced noise^44^ and reach the highest value of ~47 kHz at 1980 nm. The contribution from the FP cavity amounts to 2.6 kHz, that is sufficiently lower than the comb under investigation.

### Validation of the technique

**Figure 6:**
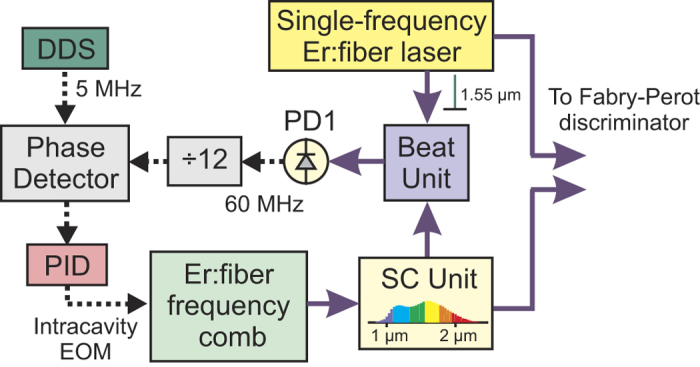
Setup for the coherence transfer from the single-frequency Er:fiber laser to the filtered comb at 1560 nm. DDS: direct digital synthesizer; SC: supercontinuum.

**Figure 7:**
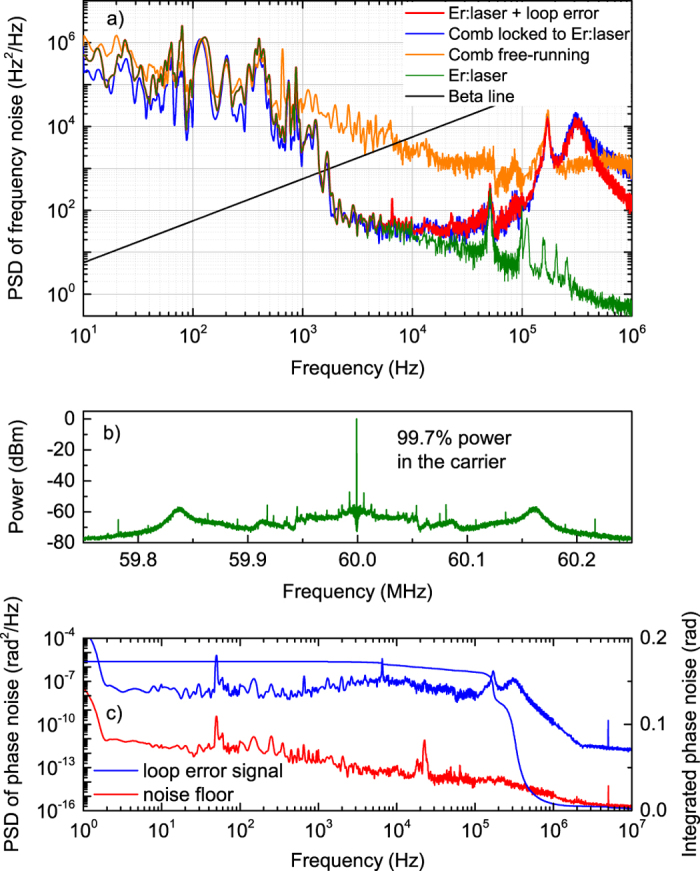
(**a**) FN PSD of the free-running comb at 1560 nm (orange), single-frequency Er:fiber laser (green), comb phase-locked to the single-frequency Er:fiber laser (blue), and PSD (red) calculated by summing the frequency noise of the loop error signal and the single-frequency Er:fiber laser. (**b**) Coherent peak of the beatnote between the comb and the single-frequency Er:fiber laser in locked conditions. (**c**) Phase noise PSD and integrated phase noise of the loop error signal.

The technique proposed for the measurement of FN of OFCs has been validated using a high-spectral purity single-frequency Er:fiber laser (SFL) at 1560 nm. A comb tooth has been phase-locked to the SFL using the setup shown in Fig. 6. In particular, the SFL and comb beams are combined in a beat-unit and focused on a low noise InGaAs photodetector. The instantaneous frequency of the resulting beating is divided by 12 using a prescaler, and then compared in a phase detector with a low-phase noise synthesizer generating a reference signal at 5 MHz. The output of the phase detector is sent to a fast proportional-integral-derivative (PID) controller acting on an intracavity electrooptic modulator and hence on the comb repetition frequency (actuating bandwidth of ~250 kHz). Figure 7b)shows the beating in locked condition, characterized by a coherent peak with SNR higher than 60 dB at 1-Hz resolution bandwidth and 99.7% of the beatnote power (250-kHz span).

The performance of the phase-lock was further characterized by measuring the PSD of the error signal at the output of the phase detector; the result is reported in Fig. 7c) together with the integrated residual phase noise. The noise floor in the phase noise measurement can be evaluated by feeding both inputs of the phase detector with the same 5-MHz reference signal from the synthesizer: in this configuration, the signals compared by the phase detector are inherently phase-coherent, hence any detected phase difference at the output of the phase detector is due to noise and represents the noise floor. The bump in the phase-noise PSD indicates a control loop bandwidth of ~250 kHz. A residual phase noise of 0.17 rad is obtained over an integration bandwidth from 1 Hz to 10 MHz, indicating that more than 97% of the RF power is concentrated in the coherence peak of the beat note signal. It’s worth to note that the PSD of the beatnote in Fig. 7b) is limited to 250 kHz, whereas the measurement of phase noise is extended up to 10 MHz; apart from the different span, the two measurements are essentially similar, however, the phase noise power resulting from integration over the larger span from 1 Hz to 10 MHz is higher and hence the percentage of RF power in the coherence peak retrieved from this measurement amounts to 97%, that is lower than the 99.7% obtained from the beatnote measurement. Under this condition, the coherence properties of the SFL are cloned on the comb teeth around 1560 nm, meaning that FN measurements of the SFL and comb, using the proposed technique, must give same results. Figure 7a) reports the measured comb FN at around 1560 nm in both the free-running (orange line) and phase-locked (blue line) conditions. The FN PSD of the SFL is also reported (green line), as measured using the same FP cavity adopted for the OFC. In locked condition, the FN PSD of the comb closely resembles that of the SFL up to 10 kHz; moreover, a strong noise reduction is observed with respect to the free-running condition up to Fourier frequencies of ~100 kHz. At frequencies larger than 200 kHz, due to the control bandwidth limitation, the FN PSD is higher than that in free-running but still below the beta-line, and does not contribute to the laser linewidth, according to the analysis reported in^41^. It’s worth noting that the bump at 180 kHz observed in the FN PSD of the FP cavity (see Fig. 5(a,b)) is not present in the FN PSD of the SFL (green line) as it is only ascribed to the locking servo acting in the cavity-stabilized Er:fiber laser.

As a final test, the FN PSD of the residual phase noise has been calculated starting from the data in Fig. 7(c), and added to the FN PSD of the SFL; the result (red line) is in close agreement with the direct measurement of the FN PSD of the comb in locked condition. This means that the discrepancy between the FN PSD of the comb and the SFL in locked condition is due to the quality of the control loop; however, apart from this point, our results prove the validity of the proposed technique to measure the FN of an OFC. The limit to the proposed technique is represented by the FN PSD of the FP cavity adopted in the setup, and more specifically, the linewidth of the FP cavity has to be well below that of the OFC over the observation time considered. The limit imposed by the FP cavity adopted in our setup is evident from Fig. 5: the linewidth of the FP cavity over 1-ms is well below that of the OFC (at all central wavelengths), hence the measurement of the FN PSD above 1 kHz can be ascribed totally to the OFC; on the other hand, the linewidth of the FP cavity over observation times longer than 1 ms increases rapidly, reaching levels comparable to that of the OFC, hence the measurement of the FN PSD at frequency below 1 kHz has some contribution (acoustic resonances) from the FP cavity, and is not accurate. The adoption of more stable FP cavity, made of monolithic low-expansion glass, would allow for the characterization of OFC with even higher spectral purity and reduced linewidth.

## Conclusion

We proposed a flexible and high-sensitivity method for the measurement of the frequency noise properties of OFCs that allows for full characterization of the OFC across its entire optical spectrum. The method represents an extension of the fringe-side locking method to OFC sources, and is based on a FP resonator used as an array of optical frequency discriminators. In principle, this approach provides high-sensitivity measurements in any spectral region from the THz to the UV by choosing proper FP mirrors for each spectral region, without using any supplementary reference laser. The capabilities of the method were tested by an accurate characterization of the FN of an Er:fiber OFC in the spectral region from 1 to 2 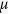
m using a FP cavity with a Finesse of ~100. We believe that this measurement technique can be easily and largely adopted for the characterization of OFC sources.

## Additional Information

**How to cite this article**: Coluccelli, N. *et al*. Frequency-noise measurements of optical frequency combs by multiple fringe-side discriminator. *Sci. Rep.* **5**, 16338; doi: 10.1038/srep16338 (2015).
